# Factors Associated With Osteoporosis Care of Men Hospitalized for Hip Fracture: A Retrospective Cohort Study

**DOI:** 10.1002/jbm4.10198

**Published:** 2019-06-27

**Authors:** Samantha L Solimeo, Kimberly McCoy, Heather Schacht Reisinger, Robert A Adler, Mary Vaughan Sarrazin

**Affiliations:** ^1^ Center for Comprehensive Access and Delivery Research and Evaluation, Iowa City VA Health Care System Iowa City IA USA; ^2^ Primary Care Data Analytics Team‐Iowa City, Iowa City VA Health Care System Iowa City IA USA; ^3^ Department of Internal Medicine Carver College of Medicine, University of Iowa Iowa City IA USA; ^4^ Hunter Holmes McGuire VA Medical Center Richmond VA USA; ^5^ Department of Internal Medicine Division of Endocrinology, Diabetes & Metabolism, Virginia Commonwealth University Richmond VA USA

**Keywords:** OSTEOPOROSIS, FRACTURE PREVENTION, FRACTURE RISK ASSESSMENT, DXA, HEALTH SERVICES RESEARCH

## Abstract

Rates of postfracture DXA and pharmacotherapy appear to be declining despite their known benefits in fracture reduction. We sought to identify factors associated with osteoporosis care among male veterans aged 50 years and older after hip fracture and to evaluate trends in rates of care with an observational cohort design using US Department of Veterans Affairs’ (VA) inpatient, pharmacy, and outpatient encounters and Centers for Medicare and Medicaid Services outpatient pharmacy claims (2007 to 2014) from men aged 50 years and older treated for hip fracture (*N* = 7317). We used the Cox proportional hazards model with random effects for the admitting facility. A sensitivity analysis was performed for a subset of patients aged 65 to 99 dually enrolled in Medicare ( *N* = 5821). Overall, approximately 13% of patients had evidence of osteoporosis care within one year of fracture. In the adjusted model, rural residence was associated with lower likelihood of care, and several comorbidities were associated with higher likelihood of receiving care. In sensitivity analyses of patients dually enrolled in Medicare, rural residence remained associated with lower likelihood of osteoporosis care. Overall rates of care decreased over time, but rates of DXA in the VA remained stable. These findings highlight the ongoing problem of low rates of postfracture care among a population with the highest risk of future fracture and its associated morbidity and mortality. The rural disparity in care and differences in rates of care across healthcare delivery systems illustrates the importance of healthcare delivery systems in promoting pharmacotherapy and DXA after sentinel events. Because the VA removes a majority of cost barriers to care, this integrated healthcare system may outperform the private sector in access to care. However, declining rates of pharmacotherapy imply knowledge gaps that undermine quality care. © 2019 The Authors. *JBMR Plus* is published by Wiley Periodicals, Inc. on behalf of American Society for Bone and Mineral Research.

## Introduction

Osteoporosis care after hip fracture should consider laboratory assessments, DXA, and pharmacotherapy.^1^, ^2^ Fall risk reduction, exercise, and nutritional strategies are of further benefit for many. The state of osteoporosis care has been recently characterized as a “perfect storm.”^3^ After a period of declining incidence, hip fracture incidence appears to be increasing.^4^ Unfortunately, not only do a majority of patients diagnosed with the condition fail to receive appropriate pharmacotherapy,^5^ a majority of those at risk of osteoporosis are not evaluated or informed of their risk, and utilization of osteoporosis‐directed therapies appears to be declining.^6^ Studies examining the prevailing low prevalence of postfracture care have repeatedly demonstrated significant differences in osteoporosis evaluation, treatment, and survival rates by gender, with men faring much more poorly than women, despite the availability of DXA and effective medications.^7^

Hospital admission for hip fracture serves as a cue to action for osteoporosis care, but opinions vary as to whether this care should be initiated during an inpatient stay by orthopedists, fracture liaison programs, or managed postdischarge through primary care.^8^, ^9^ Dedicated fracture liaison programs and orthopedics‐based interventions such as “Own the Bone” have the potential to improve the rates and timing of DXA postfracture.^10^ Such initiatives have been shown to be cost‐effective mechanisms for facilitating DXA and medication initiation, but implementation requires significant organizational commitment and coordination across laboratory, imaging, primary care, nursing, and pharmacy services.^11^, ^12^, ^13^ Similarly, adoption of primary care‐based management of osteoporosis, though patient‐centered, faces considerable and time‐consuming care coordination to monitor patients across inpatient, short‐term rehabilitation, pharmacy, and outpatient settings.^14^

The integration of these settings in the US Department of Veterans Affairs (VA), which provides medical care for more than 5 million US military veterans, offers an opportunity to better understand the role of patient and healthcare system factors contributing to men's osteoporosis care after hip fracture. Study objectives were to: (1) assess men's likelihood of receiving osteoporosis care within one year of inpatient hip fracture care; (2) identify patient factors related to the likelihood of receiving care; and (3) ascertain whether rates of osteoporosis care among VA patients follow similar trends to those documented in the private sector. The overarching goal of this research is to develop an evidence base to improve bone healthcare delivery systems.

## Materials and Methods

### Setting and data sources

This study was conducted as part of a larger research program examining osteoporosis care for men who use VA primary care services. The Institutional Review Board of the University of Iowa and the Iowa City VA Research and Development Committee approved the study prior to data acquisition and analysis. In the current analysis, we examine the frequency of and time to osteoporosis care, defined as receipt of DXA or osteoporosis pharmacotherapy, among men aged 50 years or greater treated for hip or pelvic fracture^15^ not associated with trauma, in VA inpatient settings from 2009 through 2013. The cohort was developed using national VA administrative data files for 2007 through 2014, accessed through the Corporate Data Warehouse on the VA informatics and computing infrastructure secure server network. Because many VA patients also receive care outside the VA, a sensitivity analysis was conducted on a subsample of men aged 65 and older who were dually enrolled in VA and Medicare. Data on services received by VA patients through Medicare were obtained from the VA Information Resource Center.

### Exclusion criteria

Of the 11,105 men with hip fracture, those with other bone conditions (eg, Paget osteomalacia), spinal cord injury, hospice care, or evidence of metastatic cancer in the 24 months prior to or 6 months following the inpatient encounter were excluded (*n* = 604). Exclusions were also made for those men without a VA primary care encounter in the 2 years prior to the fracture (*n* = 986) to ensure that the cohort only included patients reliant upon the VA for care. Men with evidence of prior osteoporosis care (eg, pharmacotherapy or DXA; *n* = 1315) were also excluded. The analytic cohort consisted of 7317 men admitted for hip fracture at 111 VA healthcare facilities.

### Outcomes

The primary outcome was defined as evidence of either DXA or osteoporosis pharmacotherapy occurring within 12 months of inpatient encounter for hip fracture. In the primary analysis, receipt of DXA was identified in VA inpatient and outpatient claims by International Classification of Diseases, Ninth Revision, Clinical Modification (ICD‐9‐CM) procedure codes or Current Procedural Terminology (CPT) codes. Pharmacotherapy was identified in VA pharmacy files, which include all prescriptions filled by VA pharmacies. Specific drugs included alendronate, risedronate, teriparatide, ibandronate, zoledronic acid, and raloxifene, but did not include prescriptions for testosterone or calcium or vitamin D supplements. In the sensitivity analysis, we additionally identified DXA and pharmacotherapy in Medicare claims for patients dually enrolled in the VA and Medicare.

### Covariates

Patient characteristics for multivariable models were identified, including patient sociodemographic information (eg, race, age, means category), year of fracture occurrence, rural residence, and comorbid conditions. Sociodemographic variables were defined using last available data on or before 2009. Rural residence was defined using rural‐urban commuting area (RUCA)^16^ codes assigned to patient residence zip codes, and were categorized as urban or rural. Comorbid conditions were identified using algorithms originally developed by Elixhauser based on ICD‐9‐CM diagnosis codes present on VA inpatient and outpatient claims incurred within 2 years prior to the index fracture.^17^ Additionally, dates of death were identified from the VA Vital Status file.

## Analysis

The primary outcome was receipt of either DXA or pharmacotherapy within one year of VA inpatient hip fracture encounter. An “Unknown” category was created to account for missing data in sociodemographic covariates. Unadjusted differences in patient characteristics between men who did and did not receive osteoporosis care were examined with univariate methods (ie, chi‐square or Fisher's exact where appropriate). Overall survival and survival probabilities by individual covariates were examined using Kaplan‐Meier methods, while censoring for death and end of the observation period (December 31, 2013). Subsequently, univariate and multivariate Cox proportional hazards models were used to estimate the hazard ratio (HR) relative hazard of osteoporosis care (and 95% CIs) associated with patient characteristics, while controlling for the admitting VA hospital using random effects (ie, frailty models). Variables included in the model building process were informed by literature review and clinical expertise. First, stepwise regression with the score option was used to determine the best subset of variables. Next, facility was included as a random variable to adjust for clustering. Finally, backward regression was utilized and covariates were removed iteratively, eliminating all variables that lacked significance at *p* < 0.05. As final model validation, all variables with *p* < 0.25 in the complete model were reintroduced into the final model to assess for statistical significance. No additional variables were retained.

Analyses were repeated for patients aged 65 and older who were dually enrolled in Medicare, and included DXA and pharmacotherapy received inside as well as outside the VA through Medicare. Comparison of rates of care by year was conducted using the Mantel‐Haenszel chi‐square test. Analyses were conducted using SAS Enterprise Guide version 7.1 on the VA VINCI network (SAS Institute, Inc, Cary, NC, USA).

## Results

### Patient characteristics and unadjusted rates of care

Overall, the mean age at time of fracture was 76.8 years. The majority of patients were white (75%) and lived in urban areas (69%). The most commonly occurring comorbidities included back pain (37%), diabetes (31%), and hypertension (61%). Primary analysis identified 86.8% (*n* = 6349) with no evidence of osteoporosis care during the observation period (ie, up to 12 months after fracture), 31.3% (*n* = 1988) of whom died without evidence of receiving osteoporosis care. Of those who received care, 31.6% (*n* = 306) received DXA only, 19.2% (*n* = 186) received DXA followed by pharmacotherapy, 42.0% (*n* = 407) received pharmacotherapy only, and 7.1% (*n* = 69) received pharmacotherapy followed by DXA. Among those with evidence of care the average time to care was 101.3 days (range 1 to 364, SD 97.52). In unadjusted analyses, rural residence and age at the time of fracture were associated with care; however, race and socioeconomic status (ie, VA means testing category) were not (See Table 1). Diagnoses associated with greater likelihood of receiving osteoporosis care postfracture included acquired immunodeficiency syndrome (AIDs), arthritis, back pain, and nonmetastatic cancer. See Table 2 for rates of care per 1 person‐month of follow‐up.

**Table 1:** Rate of Receiving VA Osteoporosis Care Associated With Demographic Characteristics, Unadjusted

	Total patients % of total		Number of patients receiving osteoporosis care	Rate per 1 person month of follow‐up (95% CI)
	7317	100%	968	
Race				
White	5476	74.8%	722	0.015 (0.014 to 0.016)
Black	750	10.2%	101	0.014 (0.012 to 0.018)
All other	522	7.1%	69	0.015 (0.012 to 0.019)
Missing	569	7.8%	76	0.015 (0.012 to 0.019)
Rural residence (RUCA)				
Rural	1588	21.7%	170	0.012 (0.010 to 0.014)
Urban	5054	69.1%	713	0.016 (0.015 to 0.018)
Missing	675	9.2%	85	0.014 (0.011 to 0.018)
VA means category				
Service connected	2434	33.3%	317	0.015 (0.013 to 0.017)
Low income	2792	38.2%	373	0.015 (0.013 to 0.017)
All other	2091	28.6%	278	0.015 (0.013 to 0.017)
Age				
<60	417	5.7%	52	0.012 (0.009 to 0.016)
60 to 64	837	11.4%	131	0.016 (0.013 to 0.019)
65 to 69	954	13.0%	153	0.017 (0.014 to 0.019)
70 to 74	612	8.4%	100	0.018 (0.014 to 0.021)
75 to 79	883	12.1%	134	0.017 (0.015 to 0.021)
80 to 84	1188	16.2%	166	0.017 (0.014 to 0.019)
85 to 89	1437	19.6%	160	0.014 (0.012 to 0.017)
90+	989	13.5%	72	0.010 (0.008 to 0.012)

VA = US Department of Veterans Affairs; RUCA = rural‐urban commuting area.

**Table 2:** Rate of Receiving VA Osteoporosis Care Associated With Clinical Characteristics Unadjusted (*N* = 7317)

	Overall prevalence		Number of patients with condition receiving osteoporosis care	Rate per 1 person‐month of follow‐up (95% CI)
AIDs	55	0.7%	15	0.032 (0.019 to 0.052)
Alcohol abuse	707	9.7%	117	0.017 (0.014 to 0.021)
Anemia, blood loss	68	0.9%	5	0.009 (0.004 to 0.022)
Anemia, deficiency	499	6.8%	44	0.010 (0.008 to 0.014)
Arrhythmia	2011	27.5%	219	0.013 (0.012 to 0.015)
Arthritis	149	2.0%	33	0.026 (0.019 to 0.037)
Back pain	2698	36.9%	392	0.016 (0.015 to 0.018)
Chronic obstructive Pulmonary disease	2020	27.6%	224	0.014 (0.013 to 0.016)
Coagulation disorder	269	3.7%	29	0.013 (0.009 to 0.019)
Congestive heart failure	1332	18.2%	155	0.015 (0.013 to 0.018)
Depression	1876	25.6%	247	0.015 (0.013 to 0.017)
Diabetes, complicated	1045	14.3%	134	0.015 (0.013 to 0.018)
Diabetes, uncomplicated	2283	31.2%	293	0.015 (0.013 to 0.016)
Drug abuse	297	4.1%	38	0.013 (0.010 to 0.018)
Fluid or electrolyte Disorder	1148	15.7%	125	0.013 (0.011 to 0.015)
Hypertension, complicated	575	7.9%	55	0.012 (0.009 to 0.015)
Hypertension, Uncomplicated	4455	60.9%	590	0.015 (0.014 to 0.017)
Hypothyroid	417	5.7%	47	0.014 (0.010 to 0.019)
Liver disease	354	4.8%	47	0.015 (0.012 to 0.020)
Lymphoma	79	1.1%	14	0.024 (0.014 to 0.040)
Neurological disorder	958	13.1%	109	0.013 (0.011 to 0.016)
Nonmetastatic cancer	1206	16.5%	183	0.019 (0.016 to 0.021)
Obesity	205	2.8%	34	0.017 (0.012 to 0.024)
Paralysis	116	1.6%	11	0.010 (0.006 to 0.019)
Parkinson disease	419	5.7%	52	0.016 (0.012 to 0.021)
Peptic ulcer	125	1.7%	16	0.014 (0.008 to 0.023)
Peripheral vascular disease	1179	16.1%	148	0.015 (0.012 to 0.017)
Psychosis	802	11.0%	94	0.014 (0.011 to 0.017)
Pulmonary circulatory Disorder	224	3.1%	23	0.013 (0.009 to 0.020)
Renal failure	1030	14.1%	102	0.013 (0.010 to 0.015)
Ulcer, nonbleeding	97	1.3%	13	0.015 (0.009 to 0.026)
Valvular disorder	556	7.6%	63	0.014 (0.011 to 0.017)
Weight loss	499	6.8%	60	0.015 (0.012 to 0.020)

VA = US Department of Veterans Affairs; AIDS = acquired immunodeficiency syndrome.

### Factors associated with osteoporosis care after VA inpatient hip fracture

In multivariable models that included random effects for facilities, we found a decreasing trend in receipt of any osteoporosis care (Table 3, column 1), with the relative hazard of receiving any osteoporosis care 0.73 times as high in 2013 compared with 2009 (HR 0.73; 95% CI, 0.60 to 0.90; *p* < .001). This decrease was also found in a separate analysis of pharmacotherapy receipt (Table 3, column 2), but not in an analysis of DXA (Table 3, column 3). Age and race were not significantly associated with the receipt of osteoporosis care. Patients with rural residences were significantly less likely to receive any osteoporosis care compared with their urban counterparts (HR 0.72; 95% CI, 0.61 to 0.86; *p* < .001), and this relationship persisted in separate models for pharmacotherapy and DXA outcomes. Several comorbid conditions were associated with evidence of care overall: AIDs, arthritis, back pain, and nonmetastatic cancer were associated with a greater likelihood of receiving any osteoporosis care, whereas deficiency anemia and renal disease were associated with a lower likelihood of care (Table 3). In separate models for receipt of pharmacotherapy and DXA, AIDS, arthritis, and nonmetastatic cancer were associated with a greater likelihood of receiving pharmacotherapy, whereas AIDS, arthritis, back pain, and lymphoma were associated with a greater likelihood of assessment by DXA. Renal disease and drug abuse were associated with a lower likelihood of pharmacotherapy; fluid disorder and neurological disorders were associated with a lower likelihood of DXA.

**Table 3:** Patient Characteristics Associated With Receipt of VA Osteoporosis Care in Multivariable Cox Regression Models (*N* = 7317)

	Any osteoporosis care			Medication			DXA		
	HR	95% CI	*P* value	HR	95% CI	*P* value	HR	95% CI	*P* value
Year of fracture (Ref 2009)									
2010	0.99	0.82 to 1.20	0.92	0.90	0.72 to 1.13	0.36	1.02	0.79 to 1.31	0.90
2011	0.91	0.75 to 1.11	0.34	0.90	0.72 to 1.14	0.38	0.93	0.72 to 1.21	0.58
2012	0.77	0.63 to 0.95	0.01	0.73	0.57 to 0.93	0.01	0.91	0.70 to 1.19	0.51
2013	0.73	0.60 to 0.90	0.003	0.58	0.45 to 0.76	<0.0001	0.87	0.66 to 1.13	0.30
Demographic characteristics									
Age (Ref < 60)									
60 to 64	1.36	0.99 to 1.88	0.06	1.37	0.91 to 2.06	0.13	1.51	1.00 to 2.29	0.05
65 to 69	1.41	1.03 to 1.94	0.03	1.35	0.90 to 2.01	0.15	1.77	1.18 to 2.64	0.01
70 to 74	1.51	1.07 to 2.12	0.02	1.33	0.87 to 2.05	0.19	1.72	1.12 to 2.65	0.01
75 to 79	1.47	1.06 to 2.03	0.02	1.54	1.03 to 2.30	0.04	1.38	0.91 to 2.11	0.13
80 to 84	1.39	1.01 to 1.90	0.04	1.44	0.97 to 2.13	0.07	1.20	0.79 to 1.82	0.40
85 to 89	1.14	0.83 to 1.57	0.41	1.17	0.79 to 1.74	0.44	1.02	0.67 to 1.54	0.95
90+	0.82	0.57 to 1.18	0.29	1.00	0.65 to 1.55	0.99	0.57	0.34 to 0.96	0.03
Rural to urban commuting area classification (Ref = Urban)									
Rural	0.72	0.61 to 0.86	0.0004	0.69	0.56 to 0.86	0.001	0.79	0.63 to 0.99	0.04
Missing	0.85	0.65 to 1.10	0.21	0.87	0.64 to 1.19	0.38	0.87	0.62 to 1.22	0.43
Race (Ref = White)									
Black	0.87	0.70 to 1.09	0.23	0.84	0.65 1.11	0.22	0.94	0.70 to 1.24	0.65
All others	0.98	0.73 to 1.31	0.89	0.96	0.67 to 1.36	0.80	0.91	0.61 to 1.34	0.62
Unknown	1.11	0.87 to 1.43	0.40	1.18	0.88 to 1.60	0.27	0.99	0.71 to 1.38	0.95
Comorbidities									
AIDs	2.01	1.19 to 3.40	0.01	2.51	1.40 to 4.53	0.002	2.08	1.10 to 3.95	0.02
Deficiency anemia	0.72	0.53 to 0.98	0.04						
Arthritis	1.81	1.27 to 2.58	0.0009	1.83	1.18 to 2.84	0.007	1.95	1.25 to 3.03	0.003
Back pain	1.20	1.05 to 1.36	0.0075				1.37	1.15 to 1.62	0.0003
Nonmetastatic cancer	1.25	1.06 to 1.48	0.0075	1.39	1.14 to 1.68	0.001			
Renal failure	0.79	0.64 to 0.98	0.03	0.67	0.51 to 0.87	0.003			
Drug abuse				0.52	0.32 to 0.85	0.010			
Fluid disorder							0.68	0.52 to 0.90	0.006
Lymphoma							2.06	1.09 to 3.88	0.03
Neurological disorder							0.67	0.50 to 0.90	0.007

VA = US Department of Veterans Affairs; AIDS = acquired immunodeficiency syndrome.

In sensitivity analysis of men aged 65 to 99 dually enrolled in the VA and Medicare, rural residence, and renal failure, liver disease, and weight loss were predictive of not receiving care, whereas patients with arthritis and back pain had a higher likelihood of care (Table 4).

**Table 4:** Patient Characteristics Associated With Receipt of VA or CMS Osteoporosis Care in Veterans Ages 65 to 99 (*N* = 5821)

	HR	95% CI	*P* value
Year of fracture (Ref = 2009)			
2010	0.97	0.79 to 1.20	0.76
2011	0.92	0.74 to 1.15	0.48
2012	0.73	0.58 to 0.91	0.01
2013	0.75	0.60 to 0.94	0.01
Demographic characteristics			
Age (Ref = 65 to 69)			
70 to 74	1.04	0.79 to 1.35	0.78
75 to 79	1.01	0.79 to 1.29	0.93
80 to 84	0.97	0.77 to 1.24	0.83
85 to 89	0.80	0.63 to 1.02	0.07
90+	0.55	0.41 to 0.74	<0.01
Rural to urban commuting area classification (Ref = Urban)			
Rural	0.65	0.53–0.80	<0.01
Unknown	0.91	0.68–1.22	0.53
Race (Ref = White)			
Black	0.80	0.62 to 1.04	0.10
All others	0.90	0.64 to 1.25	0.52
Unknown	1.07	0.81 to 1.41	0.64
Comorbidities			
Arthritis	1.81	1.22 to 2.67	<0.00
Back pain	1.20	1.03 to 1.38	0.02
Liver disease	0.62	0.39 to 0.99	0.04
Renal failure	0.76	0.61 to 0.95	0.02
Weight loss	0.69	0.50 to 0.95	0.02

VA = US Department of Veterans Affairs; CMS = Centers for Medicare and Medicaid Services.

### Trends in annual rates of osteoporosis care after VA inpatient hip fracture

**Figure 1:**
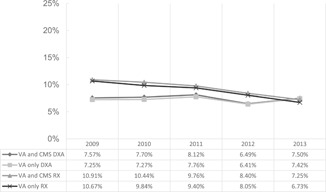
Trends in the overall percentage of men receiving osteoporosis care by year after inpatient treatment for hip fracture.

Analysis of the percentage of men receiving care after fracture identified no significant differences in DXA rates over time for veterans receiving care in VA or CMS settings. However, there was a significant downward trend in the rate of pharmacotherapy after fracture. From 2009 to 2013, rates decreased from 10.91% to 7.25% for dually eligible veterans and from 10.67% to 6.73% for veterans receiving care in the VA only (*p* < 0.001; Fig. 1).

## Discussion

In this study to identify patient factors associated with osteoporosis care after hip fracture, we determined that, given recommendations for postfracture DXA and pharmacotherapy,^1^, ^2^ men's likelihood of receiving osteoporosis care within one year of inpatient hip fracture care is substantially lower than expected. Moreover, overall receipt of osteoporosis care decreased by more than 25% over the study period. These findings confirm the low rates of care for patients at risk of osteoporosis generally, and particularly among men.^5^, ^18^, ^19^, ^20^, ^21^, ^22^ Notably, though the incidence of hip fracture in men occurs at a greater age and with far less frequency than in women, men have significantly higher postfracture mortality and morbidity, making both primary and secondary prevention efforts critical to older men's health.^23^, ^24^

We also found that osteoporosis care rates were significantly lower among rural‐residing veterans compared with urban‐residing veterans, which is consistent with other studies.^25^ Rural‐residing veterans reliant upon VA facilities for DXA may face transportation and geographic dispersion barriers as DXA is generally available in larger facilities typically located in urban communities. Access barriers to DXA may also be exacerbated by private sector trends towards consolidation of DXA services from freestanding clinics to centralized facilities.^26^ However, the recent implementation of the Veterans Access, Choice, and Accountability Act, may improve rural disparities in access to DXA by reducing travel burden and facilitating care in a veteran's home community.^27^

Several clinical factors were associated with likelihood of care, but of those that we identified as significant, HIV and renal failure are of particular interest. The relatively greater likelihood of care for those diagnosed with HIV signals appropriate identification of men at high risk of fracture.^28^, ^29^ The literature suggests that, despite guidelines for bone health evaluation of HIV‐positive men over the age of 50,^30^ healthcare providers may not be convinced of the benefits of DXA, may view bone health care as a low priority, or may lack the time to manage this condition.^30^, ^31^ The lower likelihood of pharmacotherapy among patients with renal failure may reflect known contraindications of bisphosphonates among those with impaired creatinine clearance,^32^ but the lack of association with DXA is concerning as DXA can help to quantify fracture risk in this population.^33^

Our finding that the likelihood of care overall decreased over the observation period may reflect multiple factors. We note that rates of DXA in the VA were stable over the observation period, with the decrease in care observed for pharmacotherapy only. Several have argued that after reaching peak levels in 2008 to 2009, the rates of DXA have been steadily decreasing because of reductions in CMS reimbursement and decreased access to clinic‐based DXA.^34^, ^35^, ^36^ More recently, Gillespie and colleagues demonstrated the continuation of this DXA trend for the years 2008 to 2014.^37^ The annual postfracture rates reported in our study show that, though very low, DXA rates are stable within the integrated healthcare system. Although in the private sector the low rates of care for men with hip fracture may be based in part on reimbursement or attributed to higher rates of CMS denials for male patients,^38^ such mechanisms would not apply to care in the VA, where no such gatekeeping is in place. Similarly, research examining rates of osteoporosis pharmacotherapy after fracture have documented men's lower likelihood of care as compared with women,^5^, ^19^, ^22^, ^39^ and downward trends in pharmacotherapy^22^, ^40^thought to be a result of media attention to two serious, but rare side effects—osteonecrosis of the jaw and atypical femur fracture.^39^ Rates of pharmacotherapy in our study were similar to published estimates and underscore the importance of examining this downward trend in a system designed to minimize financial and other barriers to DXA and treatment. Given that the study cohort included only those men reliant upon VA for primary care, these findings highlight the importance of efforts to provide ongoing education in bone health evaluation and management to primary care providers and to automate the identification of hip fracture patients lacking such care. The integration of clinical data across care settings in the VA's national electronic health record is a significant quality improvement resource that could be leveraged to implement secondary prevention of fracture through formal fracture liaison programs or automated clinical reminders.

Our analysis uses administrative data from a large, integrated healthcare system; however, the study is not without limitations, namely that these data do not include care provided by providers other than the VA or Medicare. Thus, it is possible that some men received care that we could not measure. Zoledronic acid coding is variable in the VA system, such that it is possible that some treatments were not captured. Although most men treated for osteoporosis in the VA are prescribed alendronate, it is possible that we are underestimating use of infusion medications within the VA. Our study examined the contribution of common comorbidities in the receipt of postfracture care, but did not incorporate all known conditions or medications associated with bone loss.

Whether one views hip fractures as sentinel events,^10^ “bone attacks,”^41^ or considers the state of osteoporosis care as “in crisis”^6^, ^35^ or “a perfect storm,”^3^ our study and others demonstrate that men are not receiving the care known to reduce their likelihood of future fractures, mortality, and related comorbidities.^1^, ^36^, ^42^, ^43^, ^44^ Within our cohort of men with hip fracture, those who are older or who reside in rural areas have a lower likelihood of care. These predictors of care represent disparities to be remedied. Although the relative stability of DXA rates over time within the VA—an integrated healthcare system that reduces most financial barriers to care— is promising, the downward trend in pharmacotherapy parallels the private sector. This downward trend in the use of medications known to reduce future fracture risk should encourage caregivers to think beyond access measures to critically examine the contributions of patients’ beliefs about osteoporosis 39, 45, 46 and healthcare providers’ clinical ownership of bone healthcare.^10^, ^47^

## Disclosures

None of the authors have any conflicts of interest.

 

## References

[1] Adler R.A. Preventing the next "bone event". J Am Geriatr Soc. 2010;58:762–64. 20398160 10.1111/j.1532-5415.2010.02778.x

[2] Camacho P.M. , Petak S.M. , Binkley N. , et al. American Asssociation of Clinical Endocrinologists and American College of Endocrinology Clinical Practice Guidelines for the Diagnosis and Treatment of Postmenopausal Osteoporosis. Endocr Pract. 2016;22 Suppl 4:1–42. 10.4158/EP161435.GL 27662240

[3] Hamdy R.C. Osteoporosis: heading towards the perfect storm. J Clin Densitom. 2018;21(1):1–2. 29325689 10.1016/j.jocd.2017.12.001

[4] Lewiecki E. , Wright N. , Curtis J. , et al. Hip fracture trends in the United States, 2002 to 2015. Osteoporos Int. 2018;29:717–22. 29282482 10.1007/s00198-017-4345-0

[5] Antonelli M. , Einstadter D. , Magrey M. Screening and treatment of osteoporosis after hip fracture: comparison of sex and race. J Clin Densitom. 2014;17(4):479–83. 24657109 10.1016/j.jocd.2014.01.009

[6] Khosla S. , Shane E. A crisis in the treatment of osteoporosis. J Bone Miner Res. 2016;31(8):1485–87. 27335158 10.1002/jbmr.2888

[7] Nayak S. , Greenspan S.L. Osteoporosis treatment efficacy for men: a systematic review and meta‐analysis. J Am Geriatr Soc. 2017;65(3):490–5. 28304090 10.1111/jgs.14668 PMC5358515

[8] Skedros J.G. , Holyoak J.D. , Pitts T.C. Knowledge and opinions of orthopaedic surgeons concerning medical evaluation and treatment of patients with osteoporotic fracture. J Bone Joint Surg Am. 2006;88A(1):18–24. 10.2106/JBJS.D.02949 16391245

[9] Simonelli C. , Killeen K. , Mehle S. , Swanson L. Barriers to osteoporosis identification and treatment among primary care physicians and orthopedic surgeons. Mayo Clin Proc. 2002;77(4):334–8. 11936928 10.4065/77.4.334

[10] Tosi L.L. , Gliklich R. , Kannan K. , et al. The American Orthopaedic Association's "own the bone" initiative to prevent secondary fractures. J Bone Joint Surg Am. 2008;90(1):163–73. 10.2106/JBJS.G.00682 18171971

[11] Ganda K. , Puech M. , Chen J.S. , et al. Models of care for the secondary prevention of osteoporotic fractures: a systematic review and meta‐analysis. Osteoporos Int. 2013;24(2):393–406. 22829395 10.1007/s00198-012-2090-y

[12] Sale J.E.M. , Beaton D. , Posen J. , et al. Systematic review on interventions to improve osteoporosis investigation and treatment in fragility fracture patients. Osteoporos Int. 2011;22(7):2067–82. 21607808 10.1007/s00198-011-1544-y

[13] Bunta A.D. , Edwards B.J. , Macaulay W.B. , et al. Own the bone, a system‐based intervention, improves osteoporosis care after fragility fractures. J Bone Joint Surg Am. 2016;98(e109):1–8. 28002377 10.2106/JBJS.15.01494 PMC5395079

[14] Seaman A. , Steffen M. , Doo T. , et al. Metasynthesis of patient attitudes toward bone densitometry. J Gen Intern Med. 2018 Oct;33(10):1796–804. 30054881 10.1007/s11606-018-4587-3 PMC6153231

[15] Colon‐Emeric C. , Pieper C.F. , Grubber J. , et al. Correlation of hip fracture with other fracture types: toward a rational composite hip fracture endpoint. Bone. 2015;81:67–71. 26151123 10.1016/j.bone.2015.07.003 PMC4772882

[16] Health Resources & Services Administration . Defining rural populations. 2017 Rockville, MD: Health Resources & Services Administration Available from: https://www.hrsa.gov/rural‐health/about‐us/definition/index.html.

[17] Elixhauser A. , Steiner C. , Harris D. , et al. Comorbidity measures for use with administrative data. Med Care. 1998;36:8–27. 9431328 10.1097/00005650-199801000-00004

[18] Liu S.K. , Munson J.C. , Bell J.E. , et al. Quality of osteoporosis care of older Medicare recipients with fragility fractures: 2006 to 2010. J Am Geriatr Soc. 2013;61(11):1855–62. 24219186 10.1111/jgs.12507 PMC4084674

[19] Jennings L.A. , Auerbach A.D. , Maselli J. , et al. Missed opportunities for osteoporosis treatment in patients hospitalized for hip fracture. J Am Geriatr Soc. 2010;58(4):650–57. 20398147 10.1111/j.1532-5415.2010.02769.x PMC2858360

[20] O'Malley C.D. , Johnston S.S. , Lenhart G. , et al. Trends in dual‐energy X‐ray absorptiometry in the United States, 2000–2009. J Clin Densitom. 2011;14(2):100–7. 21787516 10.1016/j.jocd.2011.03.003

[21] Gillespie C.W. , Morin P.E. Osteoporosis‐related health services utilization following first hip fracture among a cohort of privately‐insured women in the United States, 2008–2014: an observational study. J Bone Miner Res. 2017;32(5):1052–61. 28229485 10.1002/jbmr.3079

[22] Balasubramanian A. , Tosi L.L. , Lane J.M. , et al. Declining rates of osteoporosis management following fragility fractures in the U.S., 2000 through 2009. J Bone Joint Surg. 2014;96(7):e52.51–8. 24695929 10.2106/JBJS.L.01781

[23] von Friesendorff M. , McGuigan F. , Wizert A. , et al. Hip fracture, mortality risk, and cause of death over two decades. Osteoporos Int. 2016;27(10):2945–53. 27172936 10.1007/s00198-016-3616-5

[24] Gielen E. , Vanderschueren D. , Callewaert F. , et al. Osteoporosis in men. Best Pract Res Clin Endocrinol Metab. 2011;25(2):321–35. 21397201 10.1016/j.beem.2010.08.012

[25] Ito K. , Leslie W.D. Cost‐effectiveness of fracture prevention in rural women with limited access to dual‐energy X‐ray absorptiometry. Osteoporos Int. 2015;26(8):2111–9. 25807913 10.1007/s00198-015-3107-0

[26] Zhang J. , Delzell E. , Zhao H. , et al. Central DXA utilization shifts from office‐based to hospital‐based settings among Medicare beneficiaries in the wake of reimbursement changes. J Bone Miner Res. 2012;27(4):858–64. 22190195 10.1002/jbmr.1534

[27] Department of Veterans Affairs . Expanded access to non‐VA care through the Veterans Choice Program. Fed Regist. 2015; 80(230):74991‐6. 26634239

[28] Hoy J. Bone disease in HIV: recommendations for screening and management in the older patient. Drugs Aging. 2015;32:549–58. 26123948 10.1007/s40266-015-0279-4

[29] Gonciulea A. , Wang R. , Althoff K.N. , et al. An increased rate of fracture occurs a decade earlier in HIV + compared with HIV‐men. AIDS. 2017;31(10):1435–43. 28574962 10.1097/QAD.0000000000001493 PMC5624823

[30] Lakshmi S. , Beekmann S.E. , Polgreen P.M. , et al. HIV primary care by the infectious disease physician in the United States‐extending the continuum of care. AIDS Care. 2018;30(5):569–77. 28990409 10.1080/09540121.2017.1385720 PMC5967237

[31] Alvarez E. , Belloso W.H. , Boyd M.A. , et al. Which HIV patients should be screened for osteoporosis: an international perspective. Curr Opin HIV AIDS. 2016;11(3):268–76. 26895510 10.1097/COH.0000000000000269

[32] Miller P.D. The kidney and bisphosphonates. Bone. 2011;49(1):77–81. 21232648 10.1016/j.bone.2010.12.024

[33] Jamal S.A. , Nickolas T.L. Bone imaging and fracture risk assessment in kidney disease. Curr Osteoporos Rep. 2015;13(3):166–72. 25744703 10.1007/s11914-015-0262-3

[34] Lewiecki E.M. , Baim S. , Siris E.S. Osteoporosis care at risk in the United States. Osteoporos Int. 2008;19(11):1505–9. 18758881 10.1007/s00198-008-0716-x

[35] American Society for Bone and Mineral Research . Call to action to address the crisis in the treatment of osteoporosis 2017 Washington, DC: American Society for Bone and Mineral Research Available at: https://www.asbmr.org/call‐to‐action.aspx

[36] King A.B. , Fiorentino D.M. Medicare payment cuts for osteoporosis testing reduced use despite tests' benefit in reducing fractures. Health Aff. 2011;30(12):2362–70. 10.1377/hlthaff.2011.0233 22147865

[37] Gillespie C.W. , Morin P.E. Trends and disparities in osteoporosis screening among women in the United States, 2008–2014. Am J Med. 2017;130(3):306–16. 27884649 10.1016/j.amjmed.2016.10.018

[38] Curtis J.R. , Laster A.J. , Becker D.J. , et al. Regional variation in the denial of reimbursement for bone mineral density testing among US Medicare beneficiaries. J Clin Densitom. 2008;11(4):568–74. 18789740 10.1016/j.jocd.2008.07.004 PMC3429135

[39] Khosla S. , Cauley J.A. , Compston J.E. , et al. Addressing the crisis in the treatment of osteoporosis: a path forward. J Bone Miner Res. 2016;32(3):424–30. 28099754 10.1002/jbmr.3074

[40] Miller P.D. Underdiagnoses and undertreatment of osteoporosis: the battle to be won. J Clin Endocrinol Metab. 2016;101(3):852–9. 26909798 10.1210/jc.2015-3156

[41] Binkley N. A perspective on male osteoporosis. Best Pract Res Clin Rheumatol. 2009;23(6):755–68. 19945687 10.1016/j.berh.2009.10.001

[42] Lyles K.W. , Colón‐Emeric C.S. , Magaziner J.S. , et al. Zoledronic acid and clinical fractures and mortality after hip fracture. N Engl J Med. 2007;357(18):1799–809. 17878149 10.1056/NEJMoa074941 PMC2324066

[43] Beaupre L.A. , Morrish D.W. , Hanley D.A. , et al. Oral bisphosphonates are associated with reduced mortality after hip fracture. Osteoporos Int. 2011;22(3):983–91. 21052642 10.1007/s00198-010-1411-2

[44] Cecilia D. , Jodar E. , Fernandez C. , et al. Effect of alendronate in elderly patients after low trauma hip fracture repair. Osteoporos Int. 2009;20(6):903–10. 18956132 10.1007/s00198-008-0767-z

[45] Solimeo, S.L. Living with a 'women's disease': risk appraisal and management among men with osteoporosis. J Mens Health. 2011;8(3):185–91. 22125585 10.1016/j.jomh.2011.06.001 PMC3223980

[46] Stoecker, W.V. , Carson A. , Nguyen V.H. , et al. Addressing the crisis in the treatment of osteoporosis: better paths forward. J Bone Miner Res. 2017;32(6):1386–7. 28370446 10.1002/jbmr.3145

[47] Lee R.H. , Lyles K.W. , Pearson M. , et al. Osteoporosis screening and treatment among veterans with recent fracture after implementation of an electronic consult service. Calcif Tissue Int. 2014;94(6):659–64. 24699797 10.1007/s00223-014-9849-4 PMC4058771

